# Cultural Adaptation of CBT for Afghan Refugees in Europe: A Retrospective Evaluation

**DOI:** 10.32872/cpe.5271

**Published:** 2021-11-23

**Authors:** Schahryar Kananian, Annabelle Starck, Ulrich Stangier

**Affiliations:** 1 Department of Clinical Psychology and Psychotherapy, Goethe University Frankfurt, Frankfurt, Germany; University of Lausanne, Lausanne, Switzerland

**Keywords:** reporting criteria, cultural adaptation, Afghan refugees, transdiagnostic, group therapy

## Abstract

**Background:**

Culturally adapted CBT (CA CBT) is a well-evaluated, culture-sensitive intervention for refugees that utilizes psychoeducation, problem solving training, meditation, and stretching exercises. However, there is a lack of standard procedures for adapting psychotherapeutic interventions to a specific cultural context. Our working group adapted CA CBT for Afghan refugees at two different stages, which yielded promising results from a pilot trial and an RCT with a waitlist control group. This article aimed to illustrate the ongoing adaptation process of CA CBT for Afghan refugees over the course of several trials and to highlight potential limitations by evaluating how systematic adaptations were performed.

**Method:**

The adaptation process of CA CBT was described in detail, including the methods and rationale for changes to the protocol. This process was analyzed according to a new set of proposed reporting criteria.

**Results:**

According to the defined target population and based on multiple research strategies, culturally-specific components, such as the rationales for interventions, metaphors, and idioms of distress, were adapted. Relevant surface adaptations were implemented. However, although the steps of our adaptation process corresponded with the reporting criteria, some of the adaptation processes did not follow explicit criteria but resulted from implicit judgments.

**Conclusion:**

In the future, compliance with and the documentation of adaptation processes following explicit guidelines are crucial for the transfer of evidence-based approaches for managing the diversity of refugee populations.

## Highlights

- The reporting criteria (Heim et al., 2021, this issue) can be applied to analyze the documentation process of cultural adaptation in a post hoc analysis.
- The documentation process of culturally adapted CBT for Afghan refugees shows a high agreement with the reporting criteria.
- This detailed documentation of the adaptation process for Afghan refugees may facilitate the cultural adaptation for similar subgroups in future studies.

Approximately 18% of the refugees arriving in Germany in 2016 originated from Afghanistan. Epidemiological studies revealed high prevalence rates for PTSD (32.2%), affective disorders (21.9%), and anxiety disorders (33.9%) among Afghan refugees (Richter et al., 2015). In Afghanistan, war and armed conflicts have occurred since 1979, with only short periods of truce. However, after fleeing and seeking asylum in Western countries, distress can persist, due to long asylum procedures and restrictive housing regulations. This postmigration stress may contribute to the worsening or even development of psychopathological symptoms (Li et al., 2016; Miller & Rasmussen, 2017; Schock et al., 2016). A noticeable gap between the high prevalence rates of mental disorders and the low rates of seeking of treatments (German organization for psychotherapists [BPtK], 2015) may indicate a low acceptance and familiarity with CBT among Afghan refugees, which is also reflected by the higher dropout rates (de Haan et al., 2018). This may be related to the Western influence on CBT and how this may conflict with the values of ethnic minorities (Scorzelli & Reinke-Scorzelli, 1994).

As a low-threshold and easily accessible program, culturally adapted CBT (CA CBT), which was developed by Hinton et al. (2005), was chosen as the basic treatment concept (Hinton et al., 2005, 2009). Although other CBT interventions, as well as trauma-focused approaches, have been culturally adapted and evaluated with promising results (Hall et al., 2016; Shehadeh et al., 2016), CA CBT has been evaluated for several ethnicities, including Cambodian, Vietnamese, Egyptian, and Hispanic refugees (Hinton et al., 2005; Jalal et al., 2017). The treatment program focuses on the development of resilience, psychological flexibility, and emotional regulation. Furthermore, the group setting of CA CBT aims at overcoming the often experienced sense of isolation and in helping to establish new social networks. Finally, within a stepped care approach, CA CBT can be integrated into existing community settings and activities; thus offering the perspective to meet some of the principles that have been postulated for an ecological approach to mental health care for refugees (Miller & Rasco, 2004). As a theoretical framework for the adaptation process, we followed the guidelines by Barrera et al. (2013), which included five stages: information gathering, preliminary adaptation design, preliminary adaptation tests, adaptation refinement, and cultural adaptation trials. Although the effectiveness of cultural adaptation has been shown in several meta-analyses (Hall et al., 2016; Shehadeh et al., 2016), there is currently a lack of standardized documentation criteria. In this article, we aimed to illustrate the adaptation process of CA CBT for Afghan refugees and to depict the adaptations that were administered throughout ongoing trials by applying the criteria for reports of cultural adaptation, as suggested by Heim et al. (2021, this issue).

## Culturally Adapted CBT (CA CBT)

The program is conceptualized as being resilience-focused and subclinical, and it can be delivered in an individual or group setting and includes 14 sessions. Interventions, such as psychoeducation, stretching, meditation, guided imagery, and cognitive techniques (e.g., Socratic questioning), are a part of each session. It should be mentioned that due to its resilience-focused and transdiagnostic nature, CA CBT does not include prolonged exposure to trauma memories; instead, it focuses on emotional regulation and addresses different psychopathological symptoms, including depression, anxiety disorders, and somatic symptoms, as well as related disorders.

The original CA CBT group program by Hinton contained transcultural concepts and key idioms of distress, such as “thinking a lot” (Hinton et al., 2016), which are meant to be suitable for a variety of ethnic groups and were included in the protocol for Afghan refugees. Additionally, Hinton and colleagues (Jalal et al., 2017) adapted specific components, such as the rationales for meditation and guided imagery, for refugees from Middle Eastern Islamic cultures. The analysis of the modifications in the different protocols by Hinton and colleagues provided a blueprint of scalable components that we used to adapt the program to the Afghan culture.

## Method

### Focus Groups

Subsequently, for the pilot trial (Kananian et al., 2017), a focus group was conducted to assess the experiences of the participants. In addition, the proposed changes to the ongoing CA CBT trials were evaluated. The focus group consisted of *N* = 7 participants who had participated in the group program and whose native language was Farsi/Dari; additionally, the participants were male and over 18 years of age. The interview was conducted for approximately one hour and was audio-recorded, transcribed, and translated into German. The results were discussed by a group of experts, native speakers, and key informants. No specific qualitative analysis of the data was applied.

Experts were defined as professionals who had been working in the field of counseling psychotherapy with refugees or migrants for at least three years.

### Adaptations Following the Reporting Criteria

In the following, cultural adaptions of the CA CBT are described based on the Reporting Criteria by Heim and colleagues (2021, this issue).

#### Definition of the Target Population

At an early stage, we defined Farsi- and Dari-speaking refugees as the target population. In addition to Afghan and Syrian refugees, Iranian refugees constituted the third largest group of refugees in Germany in 2015 (Richter et al., 2015). We discussed similarities between Afghan and Iranian cultures. Although key informants raised concerns regarding potential conflicts between these two groups, due to their major differences in history and culture, many cultural similarities were recognized. This was also reflected in several articles that included Afghan and Iranian patients in a joint sample (e.g., Shishehgar et al., 2015; Steel et al., 2011). Nevertheless, throughout the group program, we identified idioms of distress that were not understood by all of the participants.

#### Cultural Concepts of Distress

##### Literature Review

We mostly derived the cultural concept of distress (CCD) for Afghan and Iranian refugees from qualitative studies that were conducted via interviews with Afghan populations (Alemi et al., 2016; Sulaiman-Hill & Thompson, 2011; Yaser et al., 2016). After a thorough review of the existing literature following idioms of distress for Farsi/Dari-speaking refugees, we included ‘asabi’ (nervous agitation), ‘gham’ (sadness), ‘jigar khun’ (a general expression of intense psychological distress), ‘tashweesh’ (worry, as proposed by Miller et al., 2006), ‘goshe-giri’ (self-isolation), ‘fekro khial’ (rumination and worrying), and ‘faramooshi’ (forgetfulness, as proposed by Alemi et al., 2016).

##### Qualitative Interviews

First, we evaluated CA CBT in individual treatments of Afghan refugees. After the treatment, we interviewed the respective patients and integrated specific suggestions into the first group manual. Many patients expressed concerns that “[they] might go crazy” and that the occurrence of the symptoms was a consequence of personal sin. Although we did not systematically analyze the qualitative data, we extended the CCD by the information that was gathered through these interviews. Further idioms of distress were identified through interviews with key informants and experts.

#### Formative Research

Although the main aspects of the adaption process in addition to the publications of the pilot trial and the RCT (Kananian et al., 2020) were reported, no additional papers on formative research were published.

#### Documenting the Decision-Making Process

We documented the statements of the experts, key informants, and native speakers who were involved in the adaptation process. Nevertheless, we did not systematically document how specific decisions were derived.

##### Team and Roles

Professor Devon E. Hinton, Associate Professor of Psychiatry, developed the original protocol of CA CBT for several ethnic groups (Hinton et al., 2005, 2009; Jalal et al., 2017).

Professor Ulrich Stangier, Professor in Clinical Psychology and Psychotherapy, who is the head of the Center for Psychotherapy and of the counseling center for refugees, as well as a supervisor and licensed psychotherapist.

Ph.D. Sarah Ayoughi, who had many years of experience in counseling in Kabul, Afghanistan, and in speaking Farsi/Dari.

##### Monitoring and Documentation

The adaptation process did not follow a documentation or monitoring methodology.

#### Diagnostics and Outcome Assessment

##### Clinical Interviews

All of the diagnostic interviews were conducted by independent Farsi-speaking postgraduate psychologists. The M.I.N.I. in the original English version (Sheehan et al., 1998) was used for the assessment, whereas key symptoms of the respective disorders were translated in advance for a more fluent and standardized assessment.

##### Questionnaires

If they were not already available and validated in Farsi, all of the instruments were translated and back-translated, in accordance with the suggested standard procedure that were proposed by van Ommeren et al. (1999).

#### Deep Structure Adaptations

##### Specific Components

*Inner Child Metaphor.* Some trauma-focused approaches to PTSD use the inner child metaphor to explain symptoms and trauma-related catastrophic cognitions (Hestbech, 2018). We did not-presume the use of this technique because it was not accepted by refugees who were individually treated. Instead, we used the metaphor of an alarm system, which was suggested to be more neutral and accessible for our specific refugee group. Nevertheless, we used the “soothing” metaphor for emotion regulation processes that were associated with the awareness of a secure environment.

*Meditation and Guided Imagery*. Due to the fact that association with positive imagery is one of the key techniques for bridging cultural barriers in psychotherapy with refugees (Hinton et al., 2005), we included guided imagery of a peaceful garden (‘bagh’) for Afghan refugees.

*Problem-Solving Training*. Inspired by treatments that were developed in programs by Rahman et al. (2016) and Sijbrandij et al. (2017), we added problem-solving training to the treatment program, which was labeled CA CBT+. Moreover, the implementation of problem-solving targets was meant to empower patients to take independent actions within their social contexts, to further their basic needs, and to broaden their socioeconomic adversities, as suggested by Miller and Rasco (2004).

##### Unspecific Components

The explanation of the treatment rationale was adapted to make it plausible and meaningful for the patients. When conveying information about the treatments to the patients, the detected CCDs and adapted specific components were taken into account to provide the treatment rationale in a culturally sensitive (e.g., values) and culturally understandable (e.g., easy language) manner. Our therapists were native speakers, which included different nonspecific components, such as a sense of belonging-and familiarity.

##### Surface Adaptations

All of the interventions (psychoeducation, problem solving training, yoga/stretching, and meditation) were explained in short written handouts in Farsi. To improve the comprehension of this information, these handouts were also audio-recorded, and both the written handouts and audiotaped information were uploaded on a website that was accessible by the participants (Stangier et al., 2020).

##### Mode of Delivery

CA CBT is available as an individual and as a group treatment. We chose a group setting for the following reasons: destigmatization through exchanges about symptoms with members of the same culture or peer group, the use of group cohesion to enhance feelings of connectedness, and dialogue about one’s experience regarding the asylum procedures.

##### Translation

Due to the fact that all of the group therapists were native speakers, no translations were required in addition to the material.

##### Matching Materials

All of the material was edited in a culturally sensitive manner and translated into Farsi/Dari via translation and retranslation. Cultural sensitivity, as for all of the other aspects of the adaptation procedure, implies the consideration of the cultural concepts of distress, gender, or religious aspects that may conflict with the values that are present in Afghan society (Eggerman & Panter-Brick, 2010). To ensure easier access to the content, audio material was prepared.

#### Documentation of Adaptations During the Trials (“on the Fly”)

Some of the on-the-fly adaptions were incorporated into the group manual (Stangier et al., 2020). For example, problem-solving training has been misunderstood as a technique for simultaneously solving all problems. The rationale for the selection of problems was explained as “picking only one stone at once from mountain”. However, we did re-evaluate on-the-fly adaptation through further discussions.

## Discussion

The examination of how we adapted CA CBT to Afghan refugees in Germany comprised a complex sequence of implicit and explicit developmental steps over three years. To analyze this process in a post hoc manner, we applied the criteria as suggested by Heim et al. (2021), this issue.

The process clearly showed that cultural adaptation contains multiple levels and aspects of an intervention, and the feedback of our interviews demonstrated its major contribution to the acceptance and, thereby, to the potentially increased effectiveness of an intervention. Due to the staged nature of cultural adaptation, even in our case of adapting an intervention that was already adapted to another culture, it remains a highly difficult and nearly impossible challenge to document all of the facets. This can be illustrated by the Farsi idiom for “thinking too much”. After the decision for the Farsi idiom “fekro khial”, we had experiences with several interpreters who suggested other translations that would be more adequate regarding the meaning of the original idiom. Throughout the varying translations of “thinking too much”, we also experienced mixed reactions from patients. The specific interpretation of the idiom that was used was dependent on the region that the patient came from. This effect demonstrated how cultural adaptation can be a meandering procedure at various times. Detailed documentation may enable other mental health professionals to profit from the thoughts, ideas, and particular adaptation steps for their own adaptation process. Our documentation shows adaptation at different steps in a way that can possibly be exemplary for other professionals. When regarding adaptation to other target groups, it may be possible to transfer or adjust adaptations, due to detailed documentation.

In the clinical context, we assume that a complete traceability of adaptations will enhance the adherence of therapists for adaptations that may seem alien to them. Furthermore, knowledge of the reasons for adaptations will increase the cultural sensitivity of mental health professionals, not only for the specific intervention, but also for the entire treatment situation. This may prevent other misunderstandings.

Nevertheless, this complexity in the communication between therapists and refugees highlights how important it is to control as much of the process as possible, in order to make the process as transparent and comprehensive as possible. Only through a standardized approach for cultural adaptation can the need for culturally sensitive psychotherapy be met. The application of explicit criteria is an important tool for establishing a standard procedure for cultural adaptations.
